# Depression comorbidity in children and adolescents with type 2 diabetes mellitus: a systematic review and meta-analysis

**DOI:** 10.3389/fendo.2026.1782080

**Published:** 2026-03-16

**Authors:** Xiaohan Wang, Shiyu You, Shihan Tang, Ying Xie, Dongmei Wu

**Affiliations:** 1 Department of Nursing, Chengdu University of Traditional Chinese Medicine, Chengdu, China; 2 Department of Nursing, Sichuan Provincial People’s Hospital, School of Medicine, University of Electronic Science and Technology of China, Chengdu, China

**Keywords:** adolescents, children, depression, prevalence, type 2 diabetes mellitus

## Abstract

**Background:**

Depression is a frequent psychological comorbidity among youth with type 2 diabetes mellitus (T2DM), yet reported prevalence rates vary widely across studies. This meta-analysis aimed to estimate the pooled prevalence of depression and explore sources of heterogeneity.

**Methods:**

This meta-analysis is registered with PROSPERO (CRD420251126166). We systematically searched PubMed, Embase, Web of Science, Cochrane Library, CINAHL, and PsycINFO from inception to October 24, 2025. The pooled prevalence was calculated using a random-effects model. Subgroup analyses and meta-regression were conducted to investigate potential sources of heterogeneity. All processes of study screening, data extraction, and quality assessment were carried out independently by two researchers.

**Results:**

Seventeen studies comprising 5,215 participants were included. The pooled prevalence of comorbid depression was 23.1% (95% CI: 18.0%–29.1%), with substantial heterogeneity (I² = 93%). Subgroup analyses indicated that prevalence differed significantly by HbA1c level and assessment method. Participants with HbA1c < 7% showed higher prevalence (52.0%) than those with HbA1c ≥ 7% (24.0%). Studies using self-report scales reported higher prevalence (25.2%) than those using clinical diagnostic criteria (12.2%). Univariate meta-regression indicated that both HbA1c level and the depression assessment method significantly influenced the reported prevalence of depression.

**Conclusions:**

Approximately one in four youth with T2DM experience depression. Variations in assessment method and glycemic control may contribute to the heterogeneity of reported prevalence. These findings underscore the importance of standardized diagnostic procedures and early psychological screening in pediatric T2DM populations to improve both mental health and diabetes outcomes.

**Systematic review registration:**

https://www.crd.york.ac.uk/prospero/, identifier CRD420251126166

## Introduction

Driven by the rising global prevalence of pediatric obesity and major lifestyle changes, the incidence of type 2 diabetes mellitus (T2DM) among children and adolescents has been increasing at an alarming rate (1, 2). Consequently, T2DM in the pediatric population has emerged as a significant global public health concern. In 2021, approximately 41,600 children and adolescents worldwide were newly diagnosed with T2DM (3). The prevalence is projected to triple by 2050 (4). This rising trend is attributed to a complex interaction among genetic susceptibility, lifestyle modifications, and other environmental factors (5). T2DM has led to adverse outcomes in this population, extending beyond its metabolic burden, such as earlier onset of complications and increased mortality rates (6–9).

Not only does T2DM confer significant metabolic risks, but it also profoundly affects mental health. Adolescence represents a critical developmental stage characterized by rapid physical, cognitive, and emotional changes (10). For adolescents with T2DM, the burden of continuous disease management is compounded by additional stressors such as academic demands, peer relationships, and identity development (11). Consequently, psychological comorbidities are common, with depression emerging as one of the most prevalent and debilitating conditions (12). Reported prevalence rates of depressive symptoms among youth with T2DM range from 14.8% to 35.5% (13). Depression can adversely affect emotional regulation, cognitive performance, and social functioning (14). Importantly, it does not occur in isolation, as shared behavioral and biological mechanisms may link depression to an increased risk of obesity, metabolic syndrome, and cardiovascular disease in youth with T2DM (15). It also impairs diabetes self-care (e.g. treatment adherence, glycemic control), creating a vicious cycle that worsens clinical outcomes and quality of life (16–19). The psychological burden extends to family members, increasing caregivers’ risk of emotional distress over time (20).

This study also includes emerging adults (21). This inclusion was adopted because emerging adulthood represents a critical transition from pediatric to adult diabetes care, marked by ongoing psychosocial challenges and developmental vulnerabilities similar to those in adolescence (22). Including this population enables a comprehensive assessment of the mental health burden in youth-onset type 2 diabetes.

Currently, there is a lack of consistent and reliable estimates of the prevalence of depression among youth with T2DM. Although numerous original studies have investigated this issue, reported prevalence rates vary substantially across studies, likely due to methodological differences and variations in study populations. Moreover, existing systematic reviews are limited by overlapping primary data and insufficient exploration of heterogeneity sources, which compromises the robustness and interpretability of their findings. Therefore, to address these gaps, we conducted a systematic review and meta-analysis to provide an updated and comprehensive estimate of the prevalence of depression among children and adolescents with T2DM, as well as to identify potential factors contributing to the observed heterogeneity across studies.

## Methods

This systematic review and meta-analysis was conducted adhering to the 2020 PRISMA guidelines. It’s also registered with PROSPERO under the registration number CRD420251126166.

### Search strategy

We systematically searched databases including PubMed, Web of Science, Embase, Cochrane Library, PsycINFO, and CINAHL, with search periods extending from each database’s inception up to October 24, 2025. To maximize inclusion of all relevant literature, the search strategy combined Medical Subject Headings terms with free-text keywords. Core search concepts included type 2 diabetes, adolescent, child, depression, and depressive symptoms. Detailed search strategies can be found in **Supplementary Table 1**.

### Study eligibility

Inclusion Criteria: (1) youth (< 24 years old) diagnosed with T2DM according to established criteria; (2) Depression was assessed using standardized instruments, validated self-reported questionnaires, or structured clinical interviews; (3) Sufficient data were reported to determine the number of depression cases or to calculate prevalence; (4) In cases of multiple publications from the same cohort, only the study with the largest sample size was included; (5) Cross-sectional studies, baseline surveys of cohort studies, or intervention trials were included. Exclusion criteria: (1) Insufficient or missing data regarding depression outcomes; (2) No English publications; (3) Articles for which the full text was unavailable; (4) Duplicate publications, theses, review articles, book chapters, or conference abstracts.

### Quality assessment

The methodological quality and risk of bias of the included studies were evaluated with the validated instrument by Hoy et al (23), which assesses ten domains of internal and external validity. Each domain was scored dichotomously: 1 point for fulfillment and 0 for non-fulfillment or uncertainty, resulting in a maximum score of 10. Subsequently, the overall risk of bias was stratified as low (>8 points), moderate (6–8 points), or high (≤5 points), consistent with the classification used in prior systematic reviews.

### Data extraction

All retrieved records were imported into EndNote reference management software. After removing duplicates, two independent reviewers (X.H.W and S.H.T) screened the studies by title, abstract, and full text. Any discrepancies were resolved through discussion, and if necessary, a third reviewer (S.Y.Y) was consulted for consensus. After finalizing the included studies, two reviewers (X.H.W and S.H.T) independently extracted data using a standardized spreadsheet (Microsoft Excel 2016). The following information was collected: first author, year of publication, study design, country, sample size, mean age, assessment tool, prevalence of depression (calculated by the reviewers if not directly reported), HbA1c level, and disease duration.

### Statistical analysis

Given the anticipated clinical and methodological heterogeneity among the included studies, a generalized linear mixed model (GLMM) was fitted to pool the prevalence estimates, in accordance with recommendations for the synthesis of proportions. This approach incorporates a random effect to account for between-study heterogeneity and uses a logit link to model the binomially distributed outcome data. Heterogeneity was quantified using the I^2^ statistic and the tau-squared (τ²) estimate derived from the Cochrane Q test, with I² values of 25%, 50%, and 75% interpreted as indicating low, moderate, and high heterogeneity (24) respectively. To explore potential sources of the heterogeneity, we performed univariate meta-regression and subgroup analysis. Univariate meta-regression was performed, primarily due to the limited number of included studies. This approach allows for a more reliable assessment of the independent association between each study-level covariate and the prevalence estimate when statistical power is low. The stability of the pooled results was evaluated through a leave-one-out sensitivity analysis. Publication bias was assessed both visually through funnel plot inspection and statistically using Egger’s and Begg’s tests. If publication bias was detected, the trim-and-fill method was applied. All statistical analyses were performed using R software (4.4.1), primarily with the meta packages.

## Results

### Study selection

**Figure 1:**
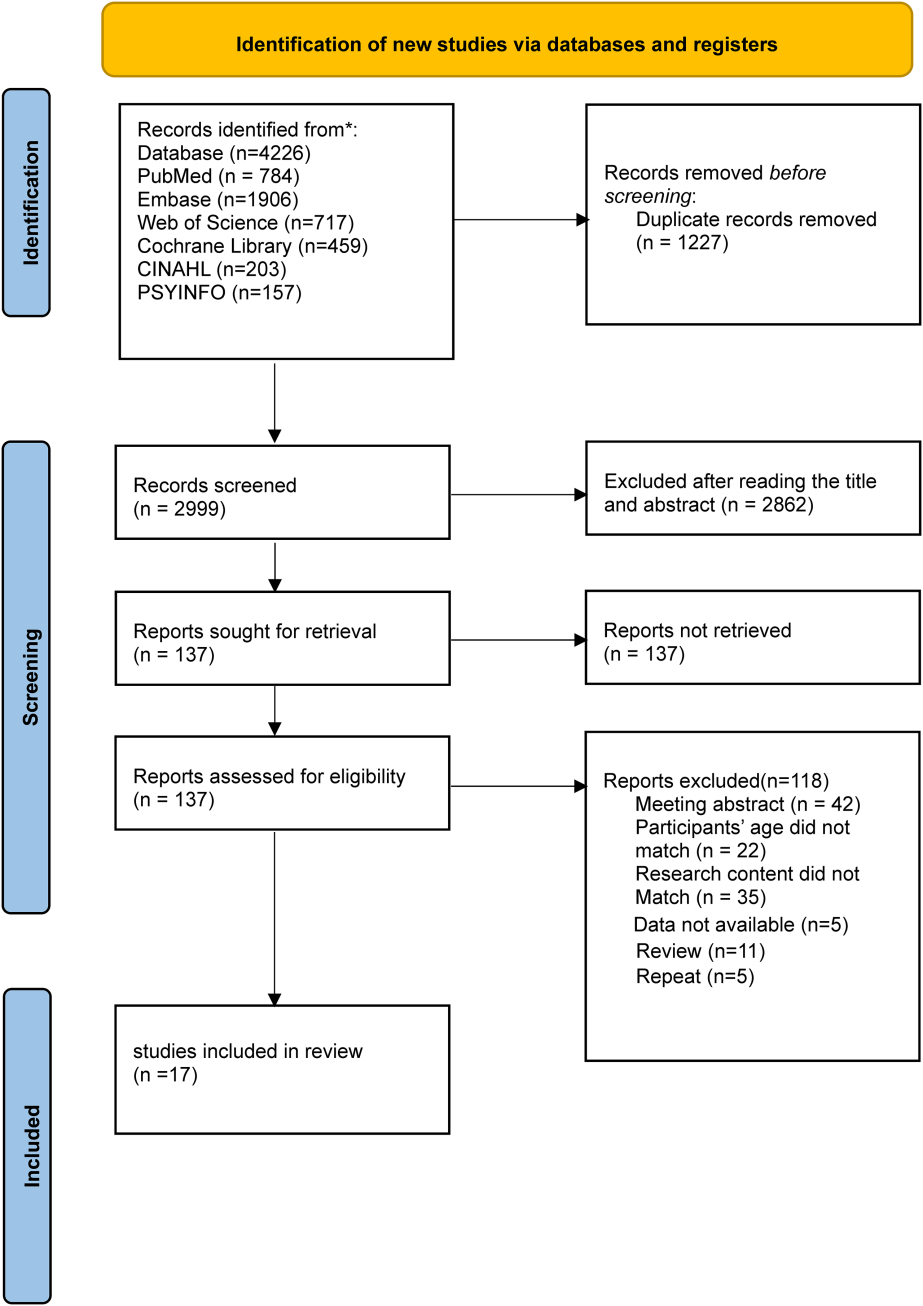
Literature screening flow diagram.

**Figure 1** illustrates the study selection process. Initially, 4226 records were gathered, and after removing 1227 duplicates, 2999 records remained. Screening the titles and abstracts led to the exclusion of 2862 records, narrowing the pool to 137 for a full-text review. Ultimately, 17 studies fulfilled the criteria for inclusion in the analysis.

### Study characteristics

**Table 1** outlines the essential characteristics of the included studies. A total of 17 studies were included, involving 5215 youth with T2DM. The participants’ ages ranged from 2 to 24 years, with the proportion of females varying from 42.4% to 100%. The majority of the studies (n=12) were cross-sectional, while the remaining studies included two randomized controlled trials, one longitudinal study, one cohort study, and one case-control study. Most of the studies were conducted in the United States (n = 15), with one each from Australia and Singapore. Among these, one study used clinical diagnostic criteria (ICD-10-CM) for diagnosing depression, while the other 16 studies assessed depression using standardized scales. Six different tools were utilized to evaluate the prevalence of depression. The most commonly used tools were PHQ-9 (n = 9), CES-D (n = 3), CDI/BDI (n = 2), HADS-D (n = 1) and PHQ-2 (n = 1).

**Table 1:** Characteristics of included studies in the systematic review and meta-analysis.

Study	Country	Design	Sample size	Age mean (SD)/range	Female (%)	Number of depression N	Methods of depression assessment/cut-off	HbA1c	Duration
Spajic 2025 (25)	Australia	cross-sectional	40	15.7 ± 2.1	55.00	23	PHQ-2 >2	6.9(6.0-9.5)	1.8(0.8–2.6)
Roy 2025 (26)	USA	cross-sectional	43	14.5 ± 1.9	53.5	12	–	–	–
Fatima 2025 (27)	USA	cross-sectional	72	16.1 ± 2.8	54.20	20	PHQ-9 >9	8.4(2.7)	2.3(1.4)
Glick 2024 (28)	USA	cross-sectional	48	13–17	52.1	12	PHQ-9 >9	–	–
Park 2024 (29)	USA	cross-sectional	2997	2–17	62.7	414	ICD-10-CM	–	–
Hoffman 2022 (30)	USA	cross-sectional	41	13–17	65.9	9	PHQ-9 >9	–	–
Zhu 2021 (31)	Singapore	longitudinal	33	19.7 ± 2.6	42.4	3	HADS-D>8	9(2.3)	4.9(4.0)
Monaghan 2021 (32)	USA	cross-sectional	197	16.9 ± 2.1	43.2	38	PHQ-9 >9	–	3.42(2.8)
Roberts 2021 (33)	USA	cross-sectional	64	15.8 ± 2.0	58	14	PHQ-9 >9	8.3(2.6)	2.6(3.5)
Wong 2020 (34)	USA	cross-sectional	27	16.5 ± 2.1	70.4	5	PHQ-9 >9	7.39(2.3)	1.92(1.7)
Picozzi 2019 (35)	USA	case-control	55	15.8	65.5	11	PHQ-9 >9	9.6(2.3)	6.3(4.2)
Glick 2018 (36)	USA	cross-sectional	63	15.4 ± 1.4	63.5	12	PHQ-9 >9	7.8(2.3)	2.2(2)
Cullum 2016 (37)	USA	cross-sectional	31	15.3 ± 1.3	61.3	11	CES-D ≥16	8.8(2.9)	2.51(2.2)
Silverstein 2015 (38)	USA	cross-sectional	339	15.1 ± 2.0	65.0	75	CDI≥13/BDI≥14	7.9(2.4)	1.7(0.6-3.3)
Lawrence 2006 (39)	USA	RCT	371	15.3 (10–21)	64.2	142	CES-D ≥16	8.4(1.9)	–
Van Buren 2018 (40)	USA	RCT	682	13.9 ± 2.0	65.5	99	CDI≥13/BDI≥14	–	–
Benson 2020 (41)	USA	cross-sectional	58	14.5	100	28	CES-D ≥16	6.7(6.0-7.1)	–

-, No report; PHQ-2, Patient Health Questionnaire-2; PHQ-9, the Patient Health Questionnaire-9; CES-D, the Center for Epidemiologic Studies Depression Scale; CDI/BDI, the Child Depression Inventory/Beck Depression Inventory; HADS-D, Hospital Anxiety and Depression Scale - Depression subscale; HbA1c: Hemoglobin A1c; RCT: randomized controlled trial.Risk of bias.

Of the 17 included studies, the majority (n = 15) were assessed as having a moderate risk of bias, primarily due to concerns regarding external validity. These concerns were mainly related to the widespread use of convenience sampling and potentially limited representativeness of the national or regional adolescent population, which may restrict the generalizability of the findings. The full quality assessment is presented in **Supplementary Table 2**.

### Meta-analysis

#### Prevalence of comorbid depression in youth with T2DM

**Figure 2:**
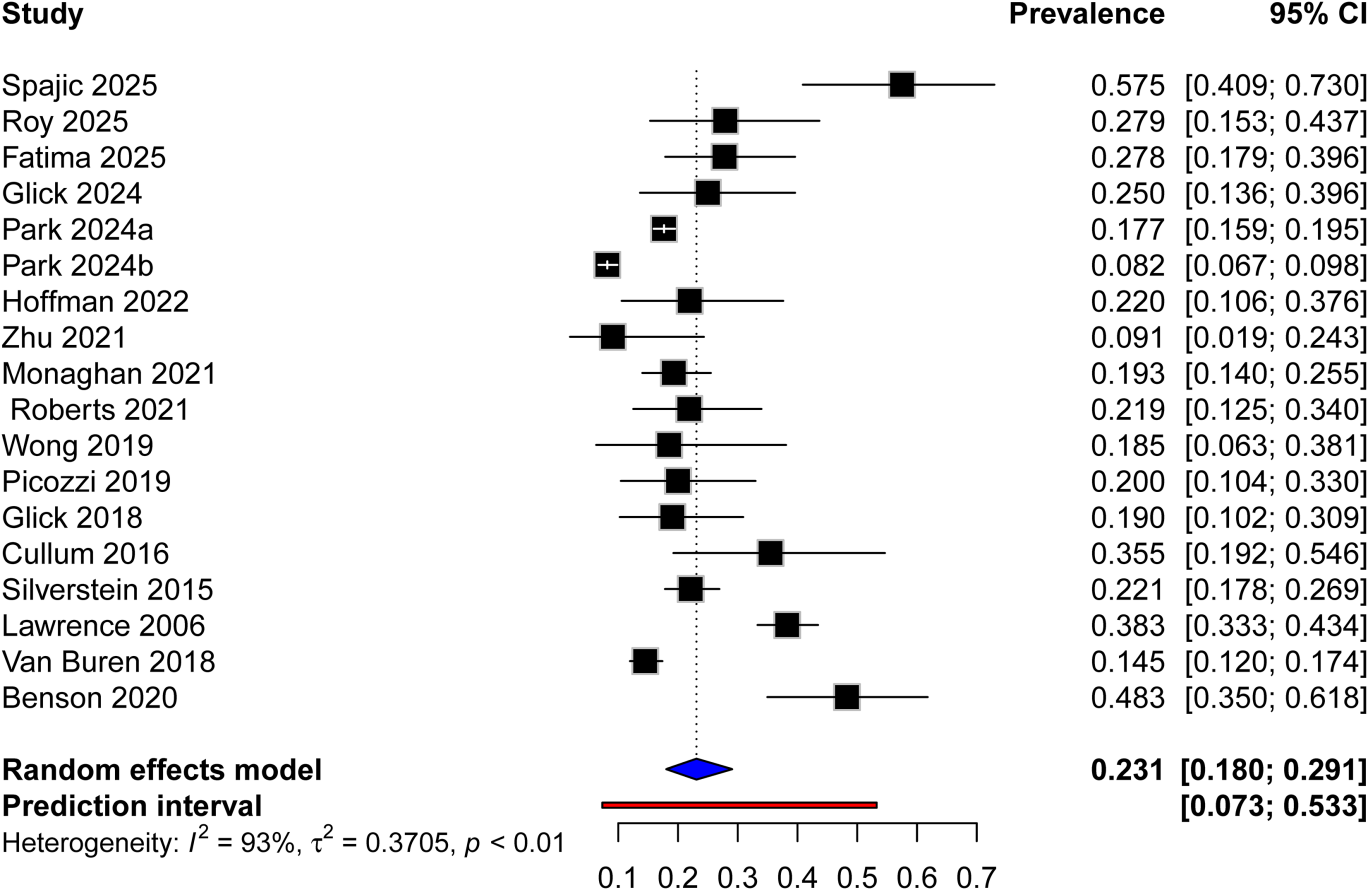
Forest plot of the prevalence of depression in child and adolescents with T2DM. a: medicaid group; b: private insurance group.

A total of seventeen studies documented the prevalence of depression among participants, with reported prevalence varying widely from 8% to 57%. A random-effects model was applied in this study, yielding a pooled prevalence of 23.1% (95% CI: 18.0%–29.1%). Substantial heterogeneity was observed among this study (I² = 93%, τ² = 0.3705) (shown in **Figure 2**).

#### Subgroup analyses of the studies

To explore potential sources of heterogeneity, subgroup analyses were performed using the following factors: sample size, depression assessment scales, HbA1c, proportion of female, and disease duration, assessment methods. In addition, univariate meta-regression analyses were performed to examine the effects of mean age, assessment method, HbA1c level, diabetes duration, and sample size on the pooled prevalence of depression. The n < 50 subgroup had a higher prevalence of depression (26.6%, 95% CI: 17.4%–38.5%) compared to the n > 50 group (21.4%, 95% CI: 15.9%–28.2%). Prevalence also varied according to HbA1c levels. Compared to the HbA1c < 7% subgroup (52%, 95% CI: 42.2%–61.7%), the HbA1c > 7% group showed a lower prevalence of 24.0% (95% CI: 18.7%–30.2%). Subgroup analysis by depression assessment tool revealed substantial differences in effect sizes between measures (*p* < 0.0001). The prevalence of depression in studies using clinical diagnostic criteria (ICD) (12.2%, 95% CI: 7.0%–20.4%) was lower than that in the self-report scale subgroup (25.2%, 95% CI: 19.7%–31.6%). Among the self-report instruments, the PHQ-2 yielded the highest pooled prevalence (57.5%, 95% CI: 42.0%–71.7%), followed by the CES-D (39.4%, 95% CI: 35.0%–43.9%). Studies using the PHQ-9 and CDI reported similar, lower prevalence estimates (21.3%, 95% CI: 18.2%–25.0%; 17.8%, 95% CI: 13.2%–23.6%). Notably, the CDI/BDI subgroup showed significant heterogeneity (I² > 89.1%). The results based on PHQ-2 (57.5%) and HADS-D (9.1%) should be interpreted cautiously, as each is derived from a single study. Regarding female ratio, studies with > 50% female participants had a higher prevalence of depression (24.3%, 95% CI: 18.7%–30.9%) than those with < 50% female participants (17.8%, 95% CI: 13.4%–23.3%) (**Supplementary Table 3**).

Univariate meta-regression analyses indicated that studies using self-report scales reported significantly higher depression prevalence compared with those employing clinical diagnostic tools (β= 0.89, 95% CI: 0.09–1.69, *p* < 0.05). In addition, sample size was negatively associated with depression prevalence (β= -0.0006, 95% CI: -0.001–0, *p* < 0.05). No significant associations were observed between depression prevalence and mean age, disease duration (**Table 2**).

**Table 2:** Univariate meta-regression analysis of pooled depression prevalence.

Variable	Number of studies	estimate (95% CI)	P	R^2^
assessment method	17	0.89(0.09,1.69)	<0.05	25.4%
Age	18	-0.07(-0.35,0.21)	=0.61	-7.7%
Sample size	18	-0.0006(-0.0011,0)	<0.05	27.4%
HbA1c	10	-0.5116(-0.85, -0.07)	<0.05	48.85%
disease duration	10	-0.19(0.43,0.04)	= 0.11	53.4%

### Sensitivity analysis and publication bias

**Figure 3:**
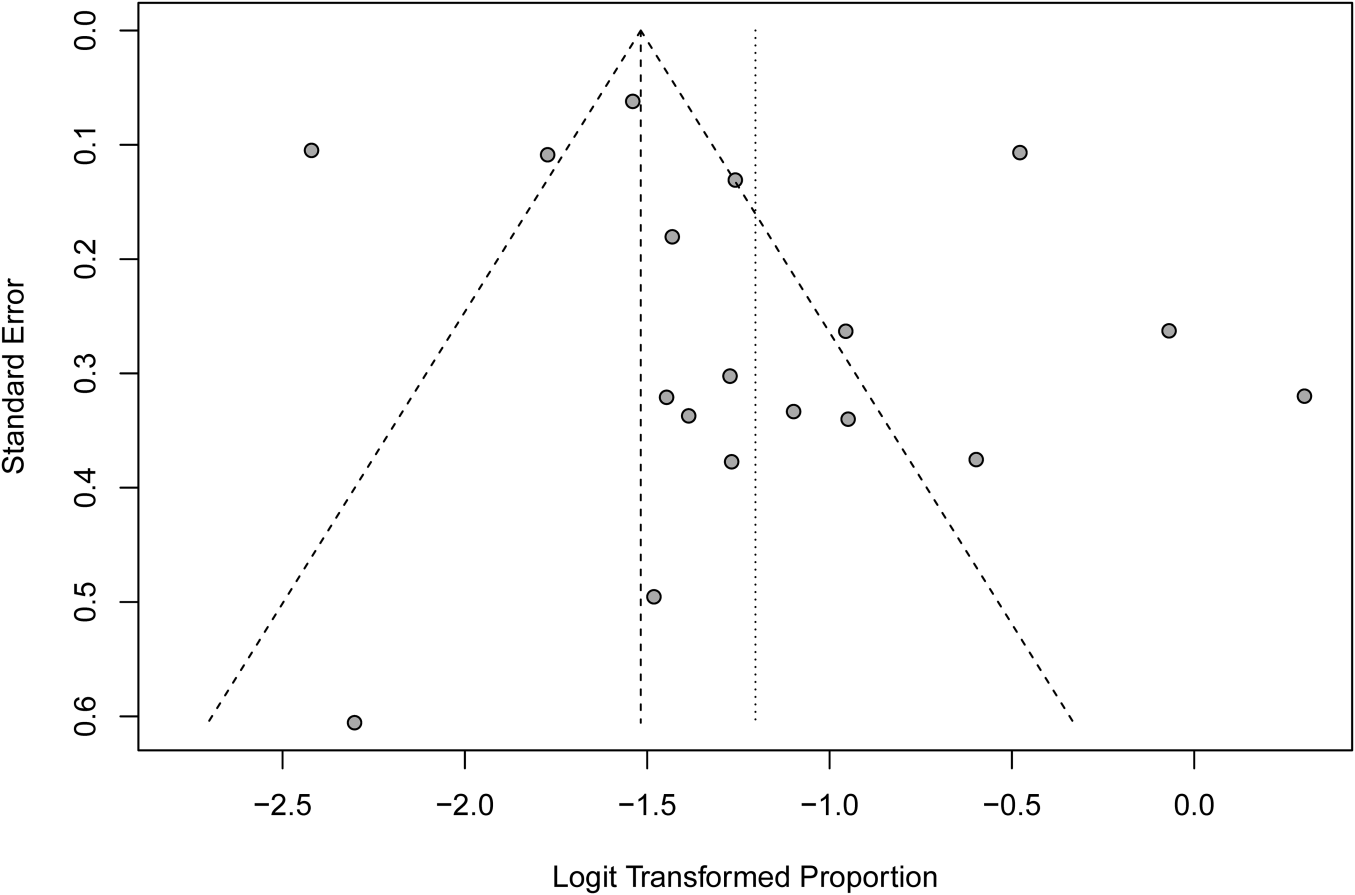
Funnel plot for the prevalence of depression in pediatric type 2 diabetes.

We conducted a sensitivity analysis using the leave-one-out approach, as illustrated in **Supplementary Figure 1**. The pooled prevalence ranged from 21.5% to 24.7%, indicating the robustness of our findings. Excluding the PHQ-2 study changed the pooled prevalence marginally from 23% to 22%. Potential publication bias was examined using funnel plots, supplemented by Egger’s test and Begg’s test. Visual inspection of the funnel plot did not reveal any evident asymmetry (**Figure 3**). Consistently, both statistical tests failed to detect significant asymmetry (Egger’s test: t = 1.28, *p* = 0.22; Begg’s test: z = 0.04, *p* = 0.97).

## Discussion

This meta-analysis synthesized data from 17 studies, involving a total of 5215 youth diagnosed with T2DM. The results indicated that nearly one in four adolescents (approximately 23.9%) experienced clinically significant depression. Subgroup analyses and univariate meta-regression further demonstrated that the prevalence of depression was influenced by the methods used for assessment and by HbA1c levels.

This study reveals that over a quarter (23.9%) of youth with T2DM experience depression, a figure that exceeds the global depression prevalence reported in both the general adolescent population and those with T1DM (42, 43). These findings indicate that depression represents a common and clinically significant comorbidity.

Social stigma and misconceptions contribute to this discrepancy. Unlike T1DM, which is widely recognized as an autoimmune disorder (44), T2DM is often erroneously attributed exclusively to obesity or unhealthy lifestyle choices. These perceptions subject adolescents with T2DM to prejudice and increased bullying risk, particularly in school settings (45–47). Many also experience a “normalization-shaming” paradox: even when attempting to minimize the significance of their condition due to its prevalence within their family or community, they struggle with internalized shame and external judgment from peers and relatives (48). Additionally, the clinical management of T2DM, which requires strict adherence to dietary restrictions, regular exercise, and medication regimens (6, 49), imposes a significant burden on adolescents, thereby exacerbating depression. During this critical developmental stage, feelings of difference and social isolation may further amplify their depression.

Besides, the distinct pathophysiology of youth-onset T2DM also contributes to the elevated risk of depression. Genome-wide association studies have identified multiple genetic loci associated with youth-onset T2DM (50), suggesting shared genetic mechanisms with psychiatric disorders (51). Separately, Wfs1 has been identified as a compelling molecular link between T2DM and mood disorders, given its established roles in insulin secretion, islet function, and energy metabolism (52–55), as well as its involvement in mood disorder susceptibility (56). Mechanistically, obesity-induced adipocyte dysfunction and inflammation not only contribute to insulin resistance but also alter brain function, increasing vulnerability to depression (57, 58). Additionally, shared HPA axis dysregulation and chronic low-grade inflammation between T2DM and depression may further exacerbate depressive symptoms (59, 60). Together, these physiological abnormalities and behavioral risk factors (e.g., low-quality nutrition, sedentary behavior, and low levels of physical activity) contribute to the development of depression in this population (61). Given the efficacy of blood transcriptomics in identifying disease-related genes and pathways (62), future studies can employ this approach to explore key genes and mechanisms in youth diabetes-depression comorbidity. These findings underscore the need for integrated care incorporating routine psychological screening, targeted interventions, and collaborative support systems involving families, schools, and healthcare providers.

It is important to note that substantial heterogeneity was observed in this study, which may be attributed to factors such as the assessment methods of depression. Studies employing self-report scales generally reported a higher prevalence of depression than those using standardized clinical diagnostic criteria (e.g., ICD-10-CM). Clinical diagnosis, while considered more specific and clinically valid, may underestimate mild or subthreshold cases of depression due to stricter diagnostic thresholds. In contrast, self-report instruments are efficient and non-invasive screening tools; however, they are more susceptible to response bias and measurement error, potentially leading to both false positives and false negatives (63–65).

The diversity of depression scales reflects the inherent heterogeneity of the disorder, which presents differently across individuals, cultural backgrounds, and social contexts (66). Additionally, these scales differ in their emphasis on affective, cognitive, and behavioral symptom domains (67).

The PHQ-9, which is based on the DSM-IV criteria for adults (68), demonstrates high specificity in identifying moderate-to-severe depressive symptoms. By adhering to these diagnostic standards, it reduces false positives and accurately identifies patients who meet the diagnostic criteria (68), resulting in a relatively conservative pooled prevalence rate of 21.3%. In contrast, the CES-D, which reported a higher prevalence of 39.4%, adopts a multidimensional structure that enhances sensitivity for detecting individuals at risk for depression (69). However, its lower specificity in distinguishing between somatic and emotional symptoms contributes to a higher false positive rate (63), potentially leading to overdiagnosis during screening. When screening adolescents with T2DM, the CDI (designed specifically for youth) may fail to capture subtle emotional fluctuations due to differences in cognitive developmental stages across age groups, increasing the risk of false negatives (63). Conversely, the BDI, originally developed for adults, struggles to address the unique, age-related depressive manifestations found in adolescents (70), thereby compromising its accuracy in this population. As a result, both the CDI and BDI report lower prevalence rates compared to the PHQ-9 and CES-D, due to these inherent limitations. These findings suggest that the level of depression an individual reports can differ widely based on the assessment tools employed. Integrating depression screening into routine diabetes care is essential. To enhance both research comparability and clinical accuracy, standardized assessment tools should be developed. These tools must distinguish between diabetes-related somatic symptoms and the core psychological features of depression, while considering the unique developmental characteristics of adolescents. Tailored approaches will improve the reliability, validity, and clinical utility of depression screening in this population.

Subgroup analysis revealed a negative relationship between HbA1c and depression prevalence among adolescents, which contrasts with the positive association reported in adults or adolescents with T1DM (71, 72). This discrepancy may be attributed to several interrelated factors. Physiologically, the discrepancy could be explained by the disease’s severity. Adolescents with T2DM frequently experience an inherent insulin deficit alongside rising insulin demand, complicating glycemic control irrespective of depression severity (61). As a result, even in the absence of severe depression, poor blood sugar management may become normalized. Psychologically, the rigorous self-management required may impose considerable emotional strain, potentially exacerbating mental health issues even as physical health improves (73). Some adolescents may also develop coping mechanisms such as denial or emotional numbing, which can lead to underreporting of depressive symptoms while maintaining focus on glycemic targets (74).

The duration of diabetes may also influence the relationship. While a new diagnosis can cause psychological distress in the early stages, residual endogenous insulin secretion often allows effective management with metformin monotherapy (75). As the disease progresses, the impact of depressive symptoms on metabolic control tends to decrease (76, 77), possibly due to improved coping and self-management skills or greater mental health support over time.

Meanwhile, socioeconomic factors also play a significant role in depression risk and metabolic control (49, 78, 79). Early access to quality healthcare services and social support can act as protective factors. However, our findings suggest a more nuanced role: In youth with T2DM, the disease itself, compounded by puberty and insulin resistance, is often inherently difficult to control. While these protective factors may not always achieve optimal glycemic management, continuous medical oversight, family support, and mental health services can effectively alleviate psychological distress and reduce depression. The absence of a direct association in some adolescent studies, underscores the complexity of these interactions (80). In conclusion, the relationship between depression and glycemic control in youth with T2DM remains incompletely understood and warrants longitudinal studies that control for confounders and evaluate integrated biopsychosocial intervention strategies.

Subgroup analysis revealed that research involving more than half female subjects exhibited a higher incidence of depression. This finding aligns with previous study (81). The TODAY investigation disclosed that women with T2DM reported a more pronounced presence of depressive symptoms compared to male (81). This observation might be attributed to adolescent hormonal changes, body fat, or physical activity. Specifically, females experience fluctuations in estrogen and progesterone during puberty. These hormones influence acute insulin response and interact with neurotransmitter systems (e.g., serotonin, dopamine), potentially increasing vulnerability to depression (2, 82). Second, studies indicated that adolescent females face a higher incidence of both obesity and T2DM than males (83), and the presence of both conditions frequently exacerbates psychological burdens. These three elements are intricately linked in a complex interplay (84, 85). Physical activity has a protective effect on both glycemic control and emotional management, while insufficient activity among females may simultaneously increase the risk of obesity, T2DM, and depression (86), partly explaining the higher prevalence of depression in female T2DM patients.

The American Diabetes Association suggests that children and adolescents with diabetes begin screening for depressive symptoms upon diagnosis, have yearly follow-up examinations, and incorporate psychosocial assessments into routine care appointments, including regular depression screening with validated tools for early detection (87). Identified cases should receive evidence-based interventions such as cognitive-behavioral therapy, psychological counseling, or support groups to address emotional distress and enhance self-management. A multidisciplinary approach should be established, with healthcare providers assessing both glycemic control and mental health during routine follow-ups to address emotional distress promptly. This helps reduce depression’s impact on treatment adherence and metabolic control, improving overall outcomes for adolescents with T2DM.

This study has several limitations. First, although subgroup analyses and meta-regressions explored multiple potential sources of heterogeneity, significant heterogeneity remained largely unexplained. Inconsistent reporting of sociodemographic (e.g., race, income) and clinical characteristics (e.g., BMI, treatment regimen) in the original studies limited further investigation of subgroup differences and may affect the stability of the pooled estimates. Second, most included studies were conducted in the United States, which may constrain the generalizability of the findings to other regions. Moreover, although this study provides overall prevalence estimates, data limitations precluded further analysis of depression symptom severity distribution. Besides, the reliance on self-report questionnaires, rather than diagnostic interviews, and the inherent false-positive rate of screening tools may have inflated depression prevalence estimates. Furthermore, significant heterogeneity in measurement instruments, populations, and settings introduced potential bias. While a random-effects model was used to mitigate this, the pooled findings should be interpreted with caution. Finally, the wide age range is a limitation, as it may mask developmental differences in depression. Future research with larger samples should employ age-stratified analyses.

Notably, this study offers the meta-analytically derived comprehensive estimate of the global burden of depression in adolescents with T2DM, establishing an evidence synthesis framework for this field. The findings highlight the necessity of integrating depression screening and psychological support into routine diabetes management, particularly in high-risk sociodemographic subgroups. Future research should adopt multicenter designs and standardized, diabetes-specific depression screening tools, control for confounders in larger samples, further validate the influence of methodological factors such as sample size, and promote the development of integrated care models that address both physical and mental health.

## Conclusion

This study reveals that over a quarter (23.9%) of youth with T2DM experience depression, underscoring its substantial impact on diabetes management and glycemic control. Despite some limitations, the findings have important public health implications, particularly in light of the concurrent global rise in both T2DM and depression among youths. These results underscore the need for regular psychological assessments following a T2DM diagnosis. Given adolescents’ vulnerability, future research must use rigorously designed prospective studies and randomized controlled trials to elucidate the bidirectional relationship between depression and diabetes, allowing for the development of effective evidence-based screening and treatment strategies.

## Data Availability

The original contributions presented in the study are included in the article/**Supplementary Material**. Further inquiries can be directed to the corresponding author.
