# Supplementary material for: Depression comorbidity in children and adolescents with type 2 diabetes mellitus: a systematic review and meta-analysis
Source: Front Endocrinol (Lausanne). 2026 Mar 16;17:1782080. doi: 10.3389/fendo.2026.1782080 (PMC13033558; doi:10.3389/fendo.2026.1782080)
Supplement: Supplementary file 1 [file DataSheet1.docx]

**Appendix. Supplementary materials**

**Title:** Depression comorbidity in children and adolescents with type 2 diabetes mellitus: A systematic review and meta-analysis

**Journal:** Frontiers in Endocrinology

**Supplementary Table 1** Full Search Strategy

| **Pubmed** |
| --- |
| #1 "Child"[Mesh] OR "Adolescent"[Mesh] |
| #2 (child[Title/Abstract] OR adolescent[Title/Abstract] OR youth*[Title/Abstract] OR teen*[Title/Abstract] OR child*[Title/Abstract] OR young adults[Title/Abstract] OR young people[Title/Abstract] OR students[Title/Abstract]) |
| #3 #1 OR #2 |
| #4 "Diabetes Mellitus, Type 2"[Mesh] |
| #5 (diabetes type 2[Title/Abstract] OR type 2 diabetes[Title/Abstract] OR non insulin dependent diabetes[Title/Abstract] OR T2DM[Title/Abstract] OR NIDDM[Title/Abstract] OR type 2 diabetes mellitus[Title/Abstract]) |
| #6 #4 OR #5 |
| #7 "Depression""[Mesh] |
| #8 (depression[Title/Abstract] OR depressive Symptoms[Title/Abstract] OR depressions[Title/Abstract] OR depression disorder[Title/Abstract] OR mental health[Title/Abstract]) |
| #9 #7 OR #8 |
| #10 #3 AND #6 AND #9 |
| **EMBASE** |
| #1. 'adolescent'/exp |
| #2. 'child'/exp |
| #3. child:ti,ab,kw OR adolescent:ti,ab,kw OR youth*:ti,ab,kw OR teen*:ti,ab,kw OR child*:ti,ab,kw OR 'young adults':ti,ab,kw OR 'young people':ti,ab,kw OR students:ti,ab,kw |
| #4. #1 OR #2 OR #3 |
| #5. 'non insulin dependent diabetes mellitus'/exp |
| #6. 'diabetes type 2':ti,ab,kw OR 'type 2 diabetes':ti,ab,kw OR 'non insulin dependent diabetes':ti,ab,kw OR t2dm:ti,ab,kw OR niddm:ti,ab,kw OR 'type 2 diabetes mellitus':ti,ab,kw |
| #7. #5 OR #6 |
| #8. 'depression'/exp |
| #9. depression:ti,ab,kw OR 'depressive symptoms':ti,ab,kw OR depressions:ti,ab,kw OR 'depression disorder':ti,ab,kw OR 'depression state':ti,ab,kw OR 'mental health':ti,ab,kw |
| #10. #8 OR #9 |
| #11 #4 AND #7 AND #10 |
| **Web of Science** |
| #1 (depression OR depressive Symptoms OR depressions OR depression disorder OR depression state OR mental health) (Title) or (depression OR depressive Symptoms OR depressions OR depression disorder OR depression state OR mental health) (Abstract) |
| #2 (child OR adolescent OR youth* OR teen* OR child* OR young adults OR young people OR students) (Title) or (child OR adolescent OR youth* OR teen* OR child* OR young adults OR young people OR students) (Abstract) |
| #3 (diabetes type 2 OR type 2 diabetes OR non insulin dependent diabetes OR T2DM OR NIDDM OR type 2 diabetes mellitus) (Title) or (diabetes type 2 OR type 2 diabetes OR non insulin dependent diabetes OR T2DM OR NIDDM OR type 2 diabetes mellitus) (Abstract) |
| #4 #1 AND #2 AND #3 |
| **Cochrane Library** |
| #1 MeSH descriptor: [Child] explode all trees |
| #2 MeSH descriptor: [Adolescent] explode all trees |
| #3 (child OR adolescent OR youth* OR teen* OR child* OR young adults OR young people OR students):ti,ab,kw |
| #4 #1 OR #2 OR #3 |
| #5 MeSH descriptor: [Diabetes Mellitus, Type 2] explode all trees |
| #6 (diabetes type 2 OR type 2 diabetes OR non insulin dependent diabetes OR T2DM OR NIDDM):ti,ab,kw |
| #7 #5 OR #6 |
| #8 MeSH descriptor: [Depression] explode all trees 19431 |
| #9 (depression OR depressive Symptoms OR depressions OR depression disorder OR mental health):ti,ab,kw |
| #10 #8 OR #9 |
| #11 (#4 AND #7 AND #10) NOT Review |
| **CINHAL** |
| #1 TI ((child OR adolescent OR youth* OR teen* OR child* OR young adults OR young people OR students)) OR AB ((child OR adolescent OR youth* OR teen* OR child* OR young adults OR young people OR students)) |
| #2 TI ((diabetes type 2 OR type 2 diabetes OR non insulin dependent diabetes OR T2DM OR NIDDM OR type 2 diabetes mellitus)) OR AB ((diabetes type 2 OR type 2 diabetes OR non insulin dependent diabetes OR T2DM OR NIDDM OR type 2 diabetes mellitus)) |
| #3 TI ((depression OR depressive Symptoms OR depressions OR depression disorder OR depression state OR mental health)) OR AB ((depression OR depressive Symptoms OR depressions OR depression disorder OR depression state OR mental health)) |
| #4 #1 AND #2 AND #3 |
| PSYINFO |
| #1 TI ((child OR adolescent OR youth* OR teen* OR child* OR young adults OR young people OR students)) OR AB ((child OR adolescent OR youth* OR teen* OR child* OR young adults OR young people OR students)) |
| #2 TI ((diabetes type 2 OR type 2 diabetes OR non insulin dependent diabetes OR T2DM OR NIDDM OR type 2 diabetes mellitus)) OR AB ((diabetes type 2 OR type 2 diabetes OR non insulin dependent diabetes OR T2DM OR NIDDM OR type 2 diabetes mellitus)) |
| #3 TI ((depression OR depressive Symptoms OR depressions OR depression disorder OR depression state OR mental health)) OR AB ((depression OR depressive Symptoms OR depressions OR depression disorder OR depression state OR mental health)) |
| #4 #1AND #2 AND #3 |

**Supplementary Table 2. Quality assessment of included studies**

| **Author & Publication Date** | **External Validity** | | | | **Internal Validity** | | | | | | **total score** | **risk of bias** |
| --- | --- | --- | --- | --- | --- | --- | --- | --- | --- | --- | --- | --- |
|  | **1** | **2** | **3** | **4** | **1** | **2** | **3** | **4** | **5** | **6** |  |  |
| Spajic 2025 | × | × | × | × | √ | √ | √ | √ | √ | √ | 6 | Moderate |
| Roy 2025 | √ | √ | √ | × | √ | √ | NA | √ | √ | √ | 8 | Moderate |
| Fatima 2025 | × | × | √ | NA | √ | √ | √ | √ | √ | √ | 7 | Moderate |
| Glick 2024 | × | × | × | √ | √ | √ | √ | √ | √ | √ | 7 | Moderate |
| Park 2024 | √ | √ | √ | √ | √ | √ | √ | √ | √ | √ | 10 | low |
| Hoffman 2022 | × | √ | × | NA | √ | √ | √ | √ | √ | √ | 7 | Moderate |
| Zhu 2021 | × | × | × | NA | √ | √ | √ | √ | √ | √ | 6 | Moderate |
| Monaghan 2021 | √ | √ | × | × | √ | × | √ | √ | √ | √ | 7 | Moderate |
| Roberts 2021 | × | × | × | NA | √ | √ | √ | √ | √ | √ | 6 | Moderate |
| Benson 2020 | × | √ | × | √ | √ | √ | √ | √ | × | √ | 7 | Moderate |
| Wong 2019 | × | √ | × | √ | √ | √ | √ | √ | √ | √ | 8 | Moderate |
| Picozzi 2019 | × | √ | × | × | √ | √ | √ | √ | × | × | 5 | high |
| Glick 2018 | × | √ | × | × | √ | √ | √ | √ | √ | × | 6 | Moderate |
| Cullum 2016 | × | √ | × | × | √ | √ | √ | √ | √ | × | 6 | Moderate |
| Silverstein 2015 | √ | √ | × | × | √ | √ | √ | √ | √ | √ | 8 | Moderate |
| Lawrence 2006 | √ | √ | × | × | √ | √ | √ | √ | √ | √ | 8 | Moderate |
| Van Buren 2018 | × | × | × | √ | √ | √ | √ | √ | √ | √ | 7 | Moderate |

External validity (maximum score=4)

1 Was the study’s target population a close representation of the national population in relation to relevant variables?

2 Was the sampling frame a true or close representation of the target population?

3 Was some form of random selection used to select the sample, OR was a census undertaken?

4 Was the likelihood of nonresponse bias minimal?

Internal validity (maximum score=6)

1 Were data collected directly from the subjects (as opposed to a proxy)?

2 Was an acceptable case definition used in the study?

3 Was the study instrument that measured the parameter of interest shown to have reliability and validity?

4 Was the same mode of data collection used for all subjects?

5 Was the length of the shortest prevalence period for the parameter of interest appropriate?

6 Were the numerator(s) and denominator(s) for the parameter of interest appropriate?

**Supplementary Table 3. Subgroup analysis of** **the prevalence of depression in children and adolescents with type 2 diabetes.**

| Variables | No. of studies | I^2^ (%) | Prevalence  （95% CI） | *P* value for subgroups |
| --- | --- | --- | --- | --- |
| Sample size |  |  |  | = 0.39 |
| <50 | 7 | 73.5 | 26.6(17.4–38.5) |  |
| >50 | 11 | 95.3 | 21.4(15.9–28.2) |  |
| Assessments scales |  |  |  | < 0.0001 |
| PHQ-9 | 8 | 0 | 21.3(18.2–25.0) |  |
| CES-D | 3 | 12.6 | 39.4(35.0–43.9) |  |
| PHQ-2 | 1 | - | 57.5(0.4–71.7) |  |
| CDI/BDI | 2 | 89.1 | 17.8(13.2–23.6) |  |
| HASD-D | 1 | - | 9.1(3-24.7) |  |
| HbA1c |  |  |  | < 0.0001 |
| <7% | 2 | 0 | 52.0(42.2–61.7) |  |
| ≥7% | 9 | 78.4 | 24.0(18.7–30.2) |  |
| Female |  |  |  | = 0.12 |
| <50% | 2 | 47.4 | 17.8(13.4–23.3) |  |
| >50% | 16 | 94 | 24.3(18.7–30.9) |  |
| assessment methods |  |  |  | < 0.05 |
| Clinical diagnosis | 2 | 98.1 | 12.2(7–20.4) |  |
| Self-reported Scale | 15 | 88.4 | 25.2(19.7–31.6) |  |

Note: -, No report; PHQ-2, Patient Health Questionnaire-2; PHQ-9, the Patient Health Questionnaire-9; CES-D, the Center for Epidemiologic Studies Depression Scale ; CDI/BDI, the Child Depression Inventory/ Beck Depression Inventory; HADS-D, Hospital Anxiety and Depression Scale - Depression subscale; HbA1c: Hemoglobin A1c.

**

**

**Supplementary Figure 1**: Sensitivity analysis of the prevalence of depression in children and adolescents with type 2 diabetes.
